# Organic Mulches as an Alternative to Conventional Under-Vine Weed Management in Mediterranean Irrigated Vineyards

**DOI:** 10.3390/plants11202785

**Published:** 2022-10-20

**Authors:** Carlos Cabrera-Pérez, Francisco Valencia-Gredilla, Aritz Royo-Esnal, Jordi Recasens

**Affiliations:** Department of Horticulture, Botany and Landscaping, ETSEA, University of Lleida, AGROTECNIO-CERCA Center, Av. Rovira Roure 191, 25198 Lleida, Spain

**Keywords:** sustainable viticulture, soil management, herbicide resistance, in-row tiller, conservation agriculture

## Abstract

Vineyard growth and grape yield can be significantly reduced by weeds, especially when these are located in the under-vine zone. Traditional weed management consists of recurrent tillage, which is associated with soil erosion and high fuel consumption, or herbicide applications, associated with damage to the environment and human health. In order to find alternative weed management methods, three field trials were carried out in Raimat (Lleida, NE Spain) with the aim of evaluating the suppressive effect of four mulches against weeds. Treatments included (1) straw mulch of *Medicago sativa* L., (2) straw mulch of *Festuca arundinacea* (L.) Schreb, (3) straw mulch of *Hordeum vulgare* L., (4) chopped pine wood mulch of *Pinus sylvestris* L., (5) mechanical cultivation and (6) herbicide application. The results showed that all mulches were efficient at controlling weeds (<20% of weed coverage) in the first year, compared with the two traditional methods, as long as the percentage of soil covered by mulches was high (>75%). In this way, pine mulch stood out above the straw mulches, as it achieved high soil cover during the three growing seasons of the study (>80%), with weed coverage values under 18%. This, together with the multiple benefits of mulches (improvements in the water balance and increases in soil organic matter, among others), make them a sustainable tool to be considered as an alternative to traditional under-vine weed management in vineyards.

## 1. Introduction

Vines are one of the most widespread crops around the Mediterranean basin, and Spain has the largest vineyard area cultivated, with 964,037 ha [1]. Vineyard growth and grape yield can be significantly reduced by weeds [2], mainly in young vines [3], as weeds compete for water, nutrients and light [4]. In most Spanish vineyards, weeds are traditionally managed through mechanical cultivation or herbicide applications in the under-vine zone [5], leaving the soil bare most of the year. The use of herbicides has been proven more effective than tillage in controlling vineyard weeds, being more cost-effective and easier to use, which has justified their use in weed management [6]. However, herbicides may cause many problems linked to environmental contamination and human health [7], a high risk of toxicity to both humans and vines [8], the potential impact of drift from commonly used auxin herbicides to leaves and grapes [9] and a reduction in root mycorrhization, which alters the nutrient composition in grapevine roots, leaves or grape juice [10]. Moreover, herbicides’ success is limited, depending on the characteristics of the weed species, the timing of the application and the weather conditions. Among the weeds that are difficult to control with herbicides in vineyards, *Conyza bonariensis* (L.) Cronquist stands out for its noxiousness [11]. It is a vigorous and competitive weed that can evolve into herbicide-resistant biotypes due to the continuous use of nonselective herbicides (e.g., glyphosate) [12]. This weed can establish at a high density in the under-vine zone, competing for water and nutrients, which can be aggravated if glyphosate-resistant biotypes are present, as these are more competitive against young vines than glyphosate-susceptible biotypes [13].

The limited lifespan of chemical tools [14] and the social demand for more sustainable agroecosystems [15] are encouraging wine growers to rethink their farming management. In fact, there are already 131,000 ha of organically managed vineyards in Spain [16], and their number is increasing each year, where weed control is recognized as the foremost production-related problem [17]. Mechanical weed management is the most common technique, which is expected to provide weed-free fields, but it has negative effects on vineyards [18,19], mainly due to damage to young vines and partly because tillage decreases the presence of grapevine roots in the topsoil [20,21]. Tillage also stimulates erosion and loss of the soil structure [22], and reduces the soil organic matter content [23,24], altering the population of soil microorganisms [25]. Furthermore, the fuel consumption of this management is double the carbon footprint of pesticides and fertilizers [26] because recurrent interventions of in-row tillering throughout the season are required to effectively manage weeds in vineyards. Moreover, cultivation brings new seeds to the surface, which, combined with an increase in soil nitrogen mineralization, leads to flushes of weed emergence [27].

In addition, repeated use of any weeding method is likely to cause a shift in the weed flora to resistant or tolerant species [28]. The integration of different weed control techniques would help to avoid this, and may provide more effective or more economic control in the current crop [29]. Mulching the under-vine zone can be an environmentally friendly useful tool for managing weeds while maintaining the grapevines’ performance and the soil’s quality. Mulch is any bulk material placed on the soil surface to control weeds and/or preserve soil moisture. Organic mulching is a sustainable agronomic practice that has an inhibitory effect on the emergence of weeds, reducing the overall weed biomass even more than the application of herbicide or cultivation [30] by creating a physical barrier for light and temperature interception [31]. Furthermore, organic mulches can cause allelopathic effects due to the substances released into the soil that can reduce the weeds’ emergence by 80% [32]. Organic mulches can also minimize water loss through evaporation [33], which is enhanced with an increase in mulch thickness [34]. Hence, the soil water content and the vines’ water status are improved [35], which can also be attributed to the proliferation of fine roots stimulated by mulches [36]. Consequently, mulches can provide substantial water savings [37]. Moreover, the infiltration of water into the soil can be also improved [38], as organic mulches increase the soil organic matter content and the soil’s biological activity [39], with a positive effect for grapevines’ yield and must composition [40]. The aim of the present work was to evaluate the weed control efficacy of four organic mulches as an alternative method to application of herbicide and mechanical cultivation, which could be incorporated in weed management programs in vineyards. In this study, the weed-suppressive effect of four mulches was evaluated, namely (1) straw mulch of *Medicago sativa* L., (2) straw mulch of *Festuca arundinacea* (L.) Schreb, (3) straw mulch of *Hordeum vulgare* L. and (4) chopped pine wood mulch of *Pinus sylvestris* L., in comparison with mechanical cultivation and applications of herbicide.

## 2. Results

### 2.1. Response of Weed Cover to Management and the Persistence of Mulch 

**Figure 1:**
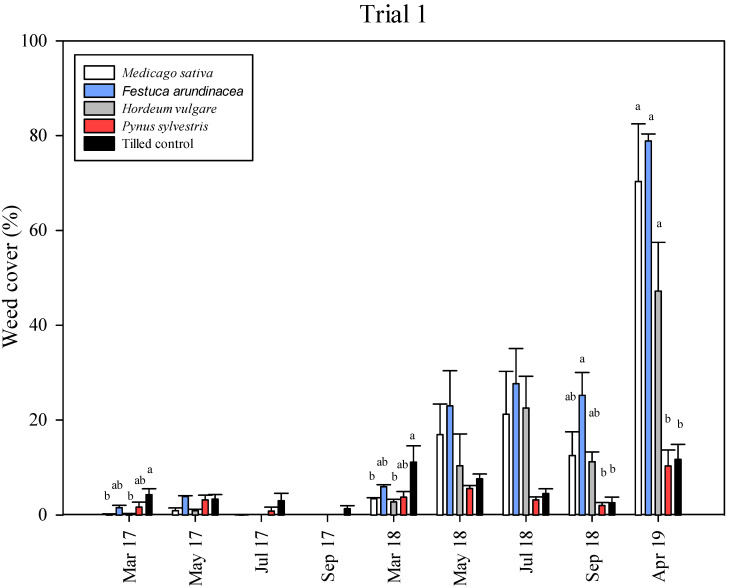
Trial 1: weed cover (%) in each treatment during the three seasons (2017, 2018 and 2019). Vertical bars represent the standard errors of the mean. Different letters indicate significant differences among treatments at *p* < 0.05.

**Figure 2:**
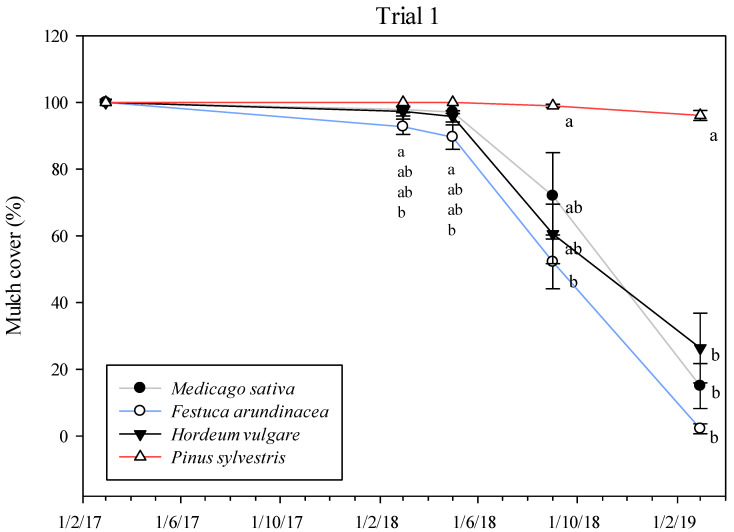
Mulch cover (%) of each mulch treatment across time in Trial 1. Vertical bars represent the standard errors of the mean. Different letters indicate significant differences among treatments at *p* < 0.05.

The overall weed cover percentage for Trial 1 was very low during the 2017 season (Figure 1), with medium values of <5% in all treatments and samples. In spring (March–May 2017), weeds were more abundant than in summer (July–September 2017). In March 2017 and 2018, the tilled control treatment had a significantly greater percentage of weed cover (Table S1, Figure 1) (4.3% and 11.2% respectively) with respect to the *M. sativa* (0.2% and 3.4%, respectively) and *H. vulgare* (0.2% and 2.6%, respectively) mulches. Conversely, from May 2018 until September 2018, the tilled control and the *P. sylvestris* mulch maintained lower a percentage of weed cover (<7%) than the straw mulches (*M. sativa*, *F. arundinacea* and *H. vulgare*), with significant differences between the both tilled control and the *P. sylvestris* mulch, and the *F. arundinacea* mulch in September 2018. In early 2019, weed coverage increased, especially in the *M. sativa* (70.3%) and *F. arundinacea* (78.9%) mulches. The lowest percentage of weed cover was observed for the *P. sylvestris* mulch (10.3%), which was significantly different from the straw mulches but not compared with the tilled control (11.7%). The persistence of the mulches (mulch cover) decreased each year, mainly in the straw ones (Figure 2), with the fastest degradation observed between 2018 to 2019, from >90% of soil covered in May 2018 for all treatments down to 2% in the *F. arundinacea* mulch, 15% in the *M. sativa* mulch and 26% in the *H. vulgare* mulch, with significant differences between *P. sylvestris* mulch and the others. In fact, *P. sylvestris* was the only mulch that remained almost unchanged, with over 95% of the soil covered by the mulch at the end of the experiment in April 2019.

**Figure 3:**
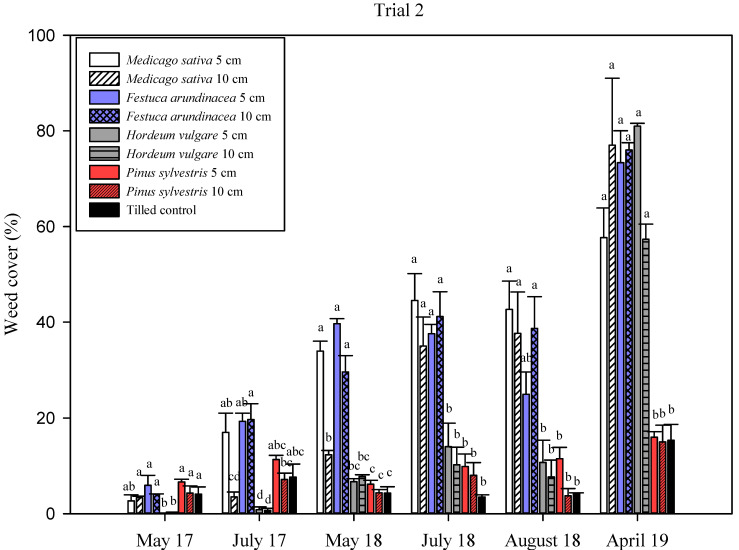
Trial 2: weed cover (%) in each treatment during the three seasons (2017, 2018 and 2019). Vertical bars represent the standard errors of the mean. Different letters indicate significant differences among treatments at *p* < 0.05.

**Figure 4:**
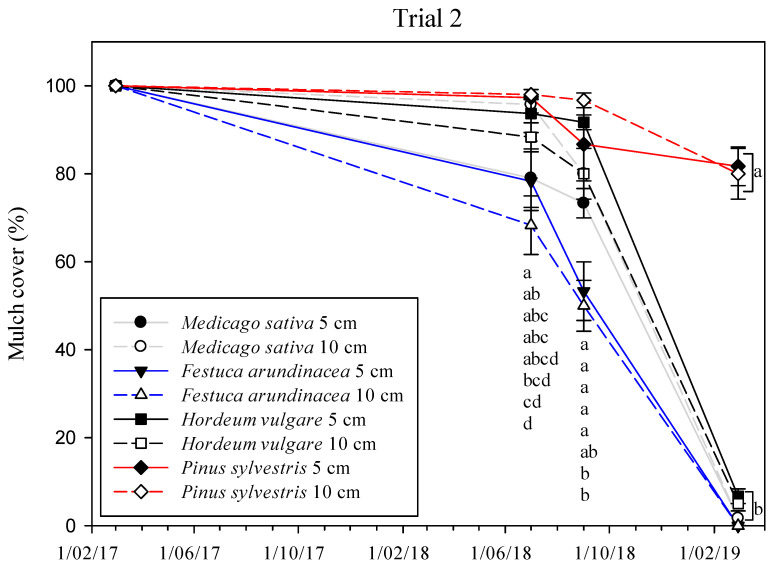
Mulch cover (%) of each mulch treatment across time in Trial 2. Vertical bars represent the standard errors of the mean. Different letters indicate significant differences among treatments at *p* < 0.05.

In Trial 2, significant differences in total weed cover were observed at each sampling date (Table S2, Figure 3). In 2017, the overall weed cover percentage was <20% for all treatments, with the *H. vulgare* mulches showing the lowest weed cover percentage (<1%) at the two thicknesses considered, and these were significantly different from the other treatments (except for *M. sativa* at 10 cm in July 2017). During 2018, only mulches of *P. sylvestris* and *H. vulgare*, and the tilled control maintained a low weed coverage (<15%), when compared with *M. sativa* and *F. arundinacea* mulches (around 40% in July 2018). In April 2019, the coverage values of the *P sylvestris* mulches and the tilled control were still low (<12%) and the differences increased with respect to the straw mulches, where percentage of cover varied from 57% up to 81%. There was no clear influence of the thickness within each mulch. The persistence of the mulches decreased similarly to those in Trial 1. The mulch cover percentage was between 0% and 7% in the straws in March 2019, and was significantly lower than in the *P. sylvestris* mulch, with >80% of the soil covered (Figure 4).

**Figure 5:**
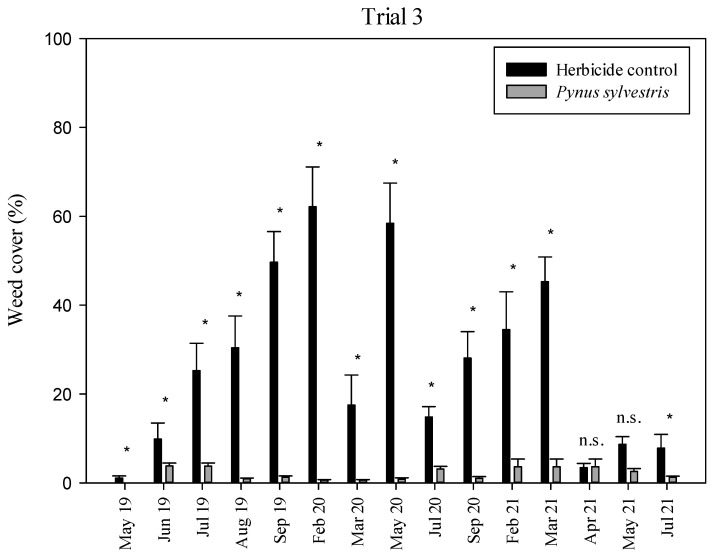
Trial 3: weed cover (%) in each treatment during the three seasons (2019, 2020 and 2021). Vertical bars represent the standard errors of the mean. * Significant differences among treatments at *p* < 0.05; n.s., not significant.

In Trial 3, weed cover values were significantly higher in the herbicide control than in the *P. sylvestris* mulch (Figure 5) from May 2019 to March 2021, and again in July 2021. During the above mentioned period, weed cover in the herbicide control was above 40% in many samplings, while by the end of 2021 season, it decreased to <10%. Conversely, weed cover in the *P. sylvestris* mulch was below 4% in 2019, 2020 and 2021 during the five samplings of each year. The *Pinus sylvestris* mulch covered the totality of the soil (100%) throughout the three seasons, and although the thickness of the mulch decreased over time, it always remained above 10 cm. The predominant species in the herbicide control was *C. bonariensis* (representing 60–85% of the total weed cover in summer), except for the last year. Winter grass species (*Hordeum murinum* L. and *Bromus rubens* L.) were also important (representing 70–90% of the total weed cover in the winter months) but only until glyphosate was applied. In the *P. sylvestris* mulch, *H. murinum* and *B. rubens* were predominant in the winter–spring months and *Convolvulus arvensis* L. was dominant during the summer months, but always with cover values of <4%.

*Conyza bonariensis* was the predominant weed in the plots treated with herbicide. The presence of this weed decreased over time, which was reflected in decreasing values of biomass, from 0.854 kg/plot in 2019 to 0.089 kg/plot in 2020 and 0.0018 kg/plot in 2021 (Table 1). The presence of this weed in the *P. sylvestris* mulch was extremely low; hence, significantly lower biomass values were obtained compared with the herbicide control in 2019 and 2020. Despite no significant differences occurring in 2021, no *C. bonariensis* plants were found in the *P. sylvestris* mulch.

### 2.2. Weed Cover and Soil Mulch Correlation

**Figure 6:**
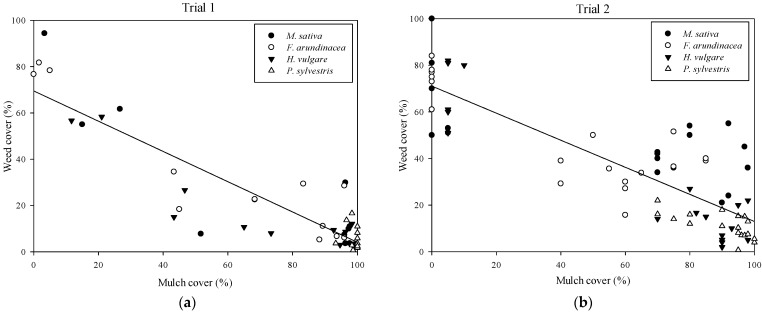
Weed cover and soil mulch correlation in (**a**) Trial 1 and (**b**) Trial 2. In Trial 2, the thicknesses have not been differentiated to facilitate interpretation. Trial 1: f = 69.5177 − 0.6538*x, R^2^ = 0.80; Trial 2: f = 71.0591 − 0.5807*x, R^2^ = 0.80.

Mulch cover was negatively related to weed cover with a linear function (*p* < 0.01; R^2^ = 0.80 and 0.71, respectively for Trials 1 and 2) (Figure 6) In Trial 2, mulch thicknesses were not considered for the graphical representation, and treatments are shown by mulch type.

### 2.3. Response of Weed Flora to Management

**Figure 7:**
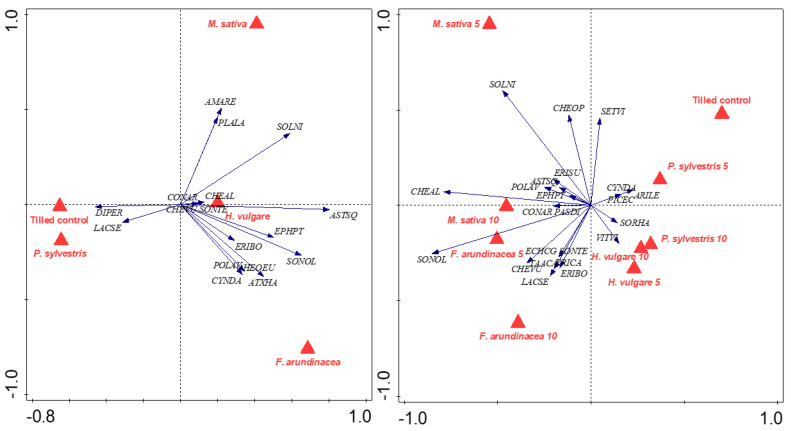
Redundancy analysis of species composition for Trial 1 (left) and Trial 2 (right). Red labels denote the treatments. Arrows show the weed species present in the analysis. Weed species are abbreviated following the EPPO Global Database, adapted from [41].

In July 2018, 17 and 23 weed species were found, respectively, in Trials 1 and 2 (Figure 7, Table 2). A permutation test showed significant variation among the treatments in Trial 1 (pseudo-F = 3.4; *p* < 0.011) and Trial 2 (pseudo-F = 2.9; *p* < 0.001). The RDA analysis explained a variance of 57.7% (49.0% and 4.7% by the first and second axis) in the weed community’s composition for Trial 1, where the analysis clearly separated the tilled control and the *P. sylvestris* mulch from the *M. sativa* and *F. arundinacea* mulches. These last mulches favored species such as *Aster squamatus* (Spreng.) Hieron., *Sonchus oleraceus* L. and *Solanum nigrum* L., among others. *Diplotaxis erucoides* (L.) DC and *Lactuca serriola* L. had some affinity for the tilled control and the *P. sylvestris* mulch, while no species was clearly related to the *H. vulgare* mulch. In Trial 2, the RDA analysis explained a variance of 55.9% (36.2% and 10.8% by the first and second axis), and the same pattern as in Trial 1 was observed, with the tilled control and the *P. sylvestris* mulch separated from the *M. sativa* and *F. arundinacea* mulches, these last two being very related to *S. oleraceus, Chenopodium album* L. and *S. nigrum*. No species could be clearly related to the tilled control or the *P. sylvestris* or *H. vulgare* mulches, and some problematic perennial species found in vineyards, such as *Cynodon dactylon* (L.) Persoon and *C. arvensis*, did not show any preferred treatment.

## 3. Discussion

Mulching has proven to be an effective strategy to control weeds in the under-vine zone. Organic mulches are known to suppress weed growth through light exclusion by creating a physical barrier [42], and through the release of allelochemicals [43,44] that may inhibit the germination of some weeds.

In Trials 1 and 2, straw mulches could maintain low rates of weeds the first season (Figure 1 and Figure 3), but an important increase in weed cover was observed during the second year, except in the *H. vulgare* mulch, where this increase was observed the third year. The *Pinus sylvestris* mulch maintained a low weed cover percentage throughout the three growing seasons, with a final weed coverage in April 2019 of only 10.3% in Trial 1 and 15% and 16% in Trial 2 for the 10 cm and 5 cm thicknesses, respectively. On the other hand, tillage was effective at maintaining an acceptable level of weed cover (<15%) when three or four mechanical interventions were performed (in 2017 and 2018), and after the first tillage in April 2019 (Figure 1 and Figure 3). However, high weed cover percentages (30–50%) preceded each tillage event, which implies high competition for resources during that period.

One of the most problematic weed species is *C. bonariensis* [11], which is very competitive against crops; nevertheless, it is easily controlled with tillage [45]. In fact, in Trial 1 and Trial 2, the presence of this species was minimized, either with tillage or with mulches, but it is difficult to control with chemical tools [46], especially when the population presents herbicide-resistant biotypes, as has been confirmed in several countries [47], Spain among them [12]. In Trial 3, the *P. sylvestris* mulch was an effective alternative in the context of glyphosate-resistant weeds such as *C. bonariensis*. Glyphosate controlled the winter–spring grass weeds but was unable to control *C. bonariensis*, which eventually developed inflorescences and disseminated achenes. In the *P. sylvestris* mulch, only residual *C. bonariensis* plants were counted in the transition zone (at the edge of the established mulch, 20 cm from the center of the under-vine zone). The total weed cover in that mulch never exceeded 5% throughout the three seasons, which clearly indicates the efficacy of this mulch for preventing the presence of weeds. Plant residues are known to decrease the germination of *C. bonariensis*, as is the case with sorghum straw [48]. The physical barrier caused by the thickness of the applied mulch might be the main reason. On one hand, the emergence of *C. bonariensis* is known to decrease with increased burial depth, and no seedling is able to emerge from deeper than 2 cm [49]. On the other hand, the absence of light produced by the mulch also decreases the germination of weeds [50].

The main weed species observed in the mulching plots was *Convolvulus arvensis*, which is a vivacious species adapted to many weed management systems and is difficult to control with straw or bark mulch, as Tebeau et al. [51] observed in their study comparing straw mulches, living mulches and tillage. However, this last method (tillage) also favors the presence of *C. arvensis*, as Abad et al. [52] reported. Conversely, Ormeño-Núñez et al. [53] observed an 82% reduction in the dry matter of *Cynodon dactylon* (another problematic rhizomatous weed species) in *Secale cereale* mulch, compared with chemical plus mechanical control, while Valencia-Gredilla et al. [54] observed that inter-row tilling and a *H. vulgare* cover crop mulched in autumn was effective in maintaining low levels of *C. dactylon* in the inter-row zone. In our study, *C. dactylon* did not show any preference for mulch or tillage treatments (Figure 7); nevertheless, this species was capable of overcoming the physical barrier of mulches and developed on top of them, probably due to its vegetative propagation thorough rhizomes.

Weed cover in spring and early summer was slightly higher than in late summer in all trials, probably due to the presence of annual winter–spring species (e.g., *D. erucoides*, *S. oleraceus* or *H. murinum*), which emerge when the soil moisture is higher and finish their life cycles before August. On the other hand, nitrophilous species such as *A. retroflexus, C. album* or *S. nigrum* were related to the *M. sativa* mulch (Figure 7), which could be associated with an increase in soil nitrate, as Teasdale and Mohler [55] observed for *T. incarnatum* residue. In these sense, Gallagher and Cardina [56] also observed that nitrate could increase the germination of *A. retroflexus* seeds.

The durability of the mulch is a key factor for achieving high weed control efficacies (Figure 6), and it is highly related to the mulch’s thickness. Bartley et al. [57], based on a one-year pot experiment, suggested 5 cm as the minimum thickness for a mulch, but they did not find differences between mulches of 5 cm and 10 cm. On the contrary, Lanini et al. [20] concluded that organic mulches need to be at least 10 cm thick to block light and be effective. The persistence of mulches at the two thicknesses considered in Trial 2 was similar over time, but the presence of weeds was more abundant in the 5 cm mulches than in the 10 cm thick mulches in most of the samplings. According to the mulch and weed cover regressions applied in Trials 1 and 2 (Figure 6), 75% to 90% mulch cover would be required to obtain 80% weed cover suppression, lower than the 97% of mulch cover predicted by Teasdale et al. [58]. The thickness of organic mulches usually declines by 60% during the first year, depending on the material [20], so most mulches need to be reapplied every two to three years. In the present study, the *P. sylvestris* mulch kept at least 80% of the soil covered for two years in Trials 1 and 2 (Figure 2 and Figure 4), and the totality of the soil (100%) after three years in Trial 3, which clearly indicates the better performance of this mulch over the other ones tested. The greater thickness of the *P. sylvestris* mulch applied in Trial 3 (15 cm) might explain the differences in the persistence of mulch between trials, together with the fact that, in Trials 1 and 2, the rows followed the slope of the ground, which was 3–4% compared with Trial 3, and may have contributed to mulch losses after rainy periods. The increase in the weeds’ presence in the straw mulches in the last season in Trials 1 and 2 (Figure 1 and Figure 2) can be explained by the fast degradation of the straw in comparison with the chopped pine wood. The large number of small particles present in the straw mulches resulted in more areas of contact with the soil, which can lead to early decomposition [59]. This is supported by Sims and Frederick [60], who found a linear relationship between early decomposition and the potential surface of straw in contact with the soil. The composition of mulches is another key factor that explains their persistence. Contrary to straw mulches, which are mainly composed of cellulose, chopped pine wood has a higher presence of lignin, which favors lower rates of decomposition [61]. Thus, straw mulches need to be reapplied every year, as would be the case for the *M. sativa* and *F. arundinacea* mulches in the present work, or every two years, in the case of the *H.* vulgare mulch. Another handicap of straw mulches is that they may have seeds incorporated in them, depending on their provenance.

Despite the low weed cover in the *P. sylvestris* mulch in all three trials, a slight increase in weed cover was observed over time, but it was much less than in the tilled control when the interventions were not frequent. There are many reports that support this, e.g., Steinmaus et al. [30] observed the higher weed control efficacy of mulching in comparison to tillage in vineyards, Fredrikson et al. [62] observed lower weed cover in their mulch treatment (an annual cover crop mix of cereal rye and *T. incarnatum* incorporated as a mulching in vineyards) when compared with mechanical cultivation, and DeVetter et al. [63] also obtained better weed control with straw and a living mulch of *Festuca rubra* L. Pennlawn than with cultivation or herbicide applications. The effectiveness of the in-row tiller depends on the frequency of interventions per year, and three to four throughout the growing season are deemed to be necessary to maintain weed cover at low levels. It is important to highlight the lack of a need for any intervention after the implementation of mulches, with the corresponding fuel savings.

Mulching represents an expensive input in vineyards. However, the amount and availability of the necessary material, and the original materials’ source, location and transport, could limit its use. Nevertheless, vineyards with organic mulch tend to suffer less thermal and water stress [64], as water losses through soil evaporation are minimized and the soil’s water holding capacity is increased in the long term due to the higher soil organic matter content [65]. A shift from traditional tillage to a mulching strategy combined with no-till practices in vineyards avoids soil compaction in the soil layers below the depth reached by the in-row tiller. Conversely, it may increase soil compaction in the upper topsoil layers in the short term and hence a deterioration in the soil’s hydrophysical properties, as Buesa et al. [35] observed when they compared both strategies in a historically tilled vineyard. In these situations, an under-vine cover crop strategy can be a useful tool, as some species can successfully compete against undesired weed species [52] while improving the soil organic matter, the soil aggregate stability and water infiltration [22,66], and avoiding compaction with the cover crop’s roots [67]. Even so, maintaining a sward under the vines could lead to lower vigor and yield in some contexts, especially in rainfed Mediterranean vineyards, while mulching generally increases them [6,68,69].

Thus, mulches have become a promising alternative for weed control in vineyards, being the most effective method compared with chemical or mechanical ones, mainly if the mulch’s persistence is beyond three years, as in *P. sylvestris* mulch, justifying the initial cost of the specialized equipment for spreading the organic material [6,70]. Despite the abovementioned benefits of mulching, they can be expensive and difficult to apply [71]. Hence, under-vine mulches may unleash their potential when addressed to specific fields with specific problems, e.g., herbicide-resistant biotypes or a high erosion risk, among others, rather than aspiring to their widespread use in vineyards.

## 4. Material and Methods

### 4.1. Experimental Site

Three field trials were established in a commercial wine grape vineyard located in Raimat (Lleida, NE Spain); the first two trials (Trials 1 and 2) were carried out from 2017 to 2019, and were located in an organically managed vineyard where the traditional under-row weed management consisted of soil cultivation with an in-row tiller with 3–4 interventions per season. Trial 3 was carried out from 2019 to 2021 in a conventional vineyard historically managed with 2–3 under-row herbicide applications per season. The field in Trial 3 had a heavy infestation of *Conyza bonariensis* in the under-vine zone (45% ± 5.2 of the soil was covered in autumn 2018). All trials were drip-irrigated regularly throughout the growing season, and the vines were trained as bilateral cordon. A spontaneous cover crop in the inter-row was shredded 2–3 times per season in all trials. The specific characteristics of the fields and vineyards are shown in Table 3. The climatic classification of this area is cold semiarid (BSk) [72], with an average annual precipitation of 342 mm and an annual mean temperature of 14.1 °C (an average minimum of 8.1 °C and an average maximum of 20.7 °C).

### 4.2. Weather Conditions

**Figure 8:**
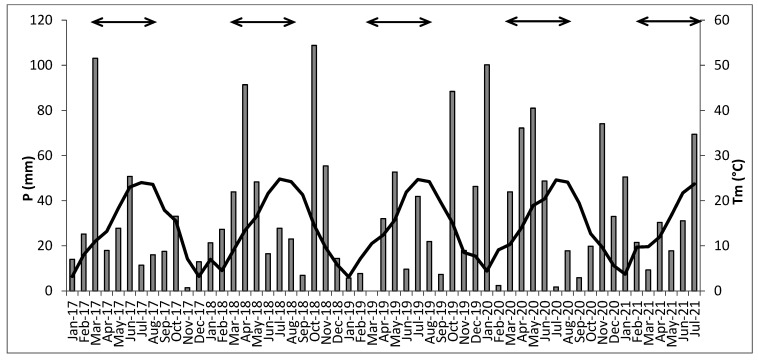
Weather conditions of the experiment period. Grey bars correspond to the total monthly precipitation (P); the black line shows the mean monthly temperature (Tm). Arrows represent the growing season each year (from March to September).

Meteorological data were obtained from an automatic weather station belonging to the regional meteorological network, located close to the vineyard in Raïmat, Ruralcat. Available online: https://ruralcat.gencat.cat/web/guest/agrometeo.estacions (accessed on 1 August 2022).The mean monthly temperature (Tm) was different among years during the growing season (black arrows in Figure 8), with 17.9 °C for 2017, 17.1 °C for 2018, 17.0 °C for 2019, 17.6 °C for 2020 and 16.8 °C for 2021, and was greatly different from the historical average (17.0 °C) in 2017 and 2020. On the other hand, 2017, 2018 and 2020 were the wettest years during the growing season, with 211 mm, 228 mm and 248 mm, respectively, and were above the historical mean (156 mm), while 2021 could be considered an average year with 158 mm, and 2019 was the driest with 136 mm of rain.

### 4.3. Experimental Design

In Trial 1, five treatments (four different mulches with no tillage and one tillage control) were established in the vineyard following a randomized complete block design with three replicates distributed in 15 rows 40 m long: (1) straw mulch of *Medicago sativa*, (2) straw mulch of *Festuca arundinacea*, (3) straw mulch of *Hordeum vulgare,* (4) chopped pine wood mulch of *Pinus sylvestris* and (5) mechanical cultivation (tillage). The mulches were applied along the under-row at 0.4 m wide and 10 cm thick. The experimental units were the average of 3 plots comprising 3 m of each row. In Trial 2, the mulches used in Trial 1 were established at two different thicknesses, 5 and 10 cm, while the tillage was again considered as the control. In this second trial, nine treatments were established, following a complete randomized design distributed over 9 rows 35 m long, with three 0.4 × 3 m replicates each. Three mechanical interventions were used in the control treatment (tillage) in both trials in the first season, four during the second season and one in the third season before the last sampling, always between February (BBCH 01) and September (BBCH 91). The mulches were applied with a vineyard manure spreader only at the beginning of the experiment, in March 2017, with no further interventions until the end of the experiment in April 2019.

In Trial 3, a complete randomized design was established over six rows with two treatments, namely (1) chopped pine wood mulch of *P. sylvestris* with no tillage, and (2) herbicide applications, each replicated six times. The experimental units were 12 plots of 0.4 m wide x 6 m long. The herbicide applied was glyphosate at 360 g a.i. L^−1^ (Roundup; Bayer CropScience, S.L., Valencia, Spain), which was applied with a manual backpack sprayer (Matabi) from a distance of 50 cm above the ground; the herbicide was applied twice in 2019 (May and June) and in 2020 (March and May), and once in 2021 (March) at 3 L/ha, always in the morning, without wind. The mulch was installed only at the beginning of the experiment, in January 2019, and was 15 cm thick. All vines within each trial were irrigated with the same amount of water and fertilized according to the standard practice of the farm. No further action (tillage, mowing, or herbicide application) was taken in the mulched plots.

### 4.4. Weed and Mulch Sampling

Weed cover was evaluated three to four times each year, except in 2019 in Trials 1 and 2, where only one sampling was performed at the beginning of the third season, in April. The total weed cover of each species was visually estimated as the percentage of the whole plot, in the case of Trials 2 and 3, or as the mean of the three subplots in the case of Trial 1. Samplings were taken after each mechanical intervention in Trials 1 and 2. In Trial 3, samplings were carried out independently of the herbicide application. The total aerial biomass of *C. bonariensis* was collected from the whole plot in each treatment every year in September, oven-dried at 67 °C for 72 h and weighted with a precision weight. The persistence of mulch was also visually estimated every season as a percentage of soil cover.

### 4.5. Statistical Analyses

The weed and mulch cover data of Trial 1 and 2 were subjected to one-way ANOVA for each sampling date, followed by multiple comparisons of the treatment effects with Tukey’s HSD-test (*p* < 0.05). When necessary, the data were square-root-transformed to meet the assumptions of ANOVA, normality (Shapiro–Wilk) and homoscedasticity (Levene’s test). The data were back-transformed for clarity in the results. To analyze the total weed cover data and biomass of *C. bonariensis* in Trial 3, the Mann–Whitney rank sum test was applied due to the impossibility of meeting the assumptions of the ANOVA. Analyses and graphs were performed with JMP Pro 15 (SAS Institute, 2010, SAS Campus Drive, Cary, NC27513, USA) and SigmaPlot 12.0 (Systat Software, San José, CA, USA). In order to evaluate the differences in the composition of weed species among the different managements, a redundancy analysis (RDA) was performed for Trials 1 and 2 with CANOCO 5.0 (Microcomputer Power: Ithaca, NY, USA, 2012).

## 5. Conclusions

Organic mulches achieve better weed control than traditional tillage or herbicide applications, as long as their persistence and soil cover are high (above 75%). Depending on the nature of the mulch, the patterns of decomposition are unequal, with the straw mulches being less persistent. THE chopped pine wood mulch stands out above the straw mulches, as it achieved high soil cover during at least three growing seasons, avoiding weed growth and the need for any other soil intervention. This, together with the multiple benefits of mulches, makes them a sustainable tool to incorporate in weed management programs in vineyards.

## Figures and Tables

**Table 1:** Total biomass (g/plot) of each treatment during the three seasons (2019, 2020 and 2021). Mean values ± standard errors of the mean.

Trial 3	Dry Weight Biomass of *C. bonariensis* (kg/plot)		
	2019	2020	2021
*Pinus sylvestris* mulch	0 ± 0	0.7 ± 0.5	0 ± 0
Herbicide control	0.854 ± 0.12	0.089 ± 0.022	0.0018 ± 0.00006
Mann–Whitney rank sum test	*p* = 0.002 *	*p* = 0.002 *	*p* = 0.065

* Significant at *p* < 0.05.

**Table 2:** Plant species analyzed in Figure 7.

EPPO Code	Scientific Name	EPPO Code	Scientific Name
AMARE	*Amaranthus retroflexus*	HEOEU	*Heliotropium europaeum*
ARILE	*Arenaria leptocloa*	LACSE	*Lactuca serriola*
ASTSQ	*Aster squamatus*	PASDI	*Paspalum dilatatum*
ATXHA	*Atriplex prostrata*	PICEC	*Picris echioides*
CHEAL	*Chenopodium album*	PLALA	*Plantago lanceolata*
CHEOP	*Chenopodium album*	POLAV	*Polygonum aviculare*
CHEVU	*Chenopodium vulvaria*	SETVI	*Setaria viridis*
CONAR	*Convolvulus arvensis*	SOLNI	*Solanum nigrum*
CYNDA	*Cynodon dactylon*	SONOL	*Sonchus oleraceus*
DIPER	*Diplotaxis erucoides*	SONTE	*Sonchus terrenimus*
ECHCG	*Echinochloa crus-galli*	SORHA	*Sorghum halepense*
EPHPT	*Euphorbia prostrata*	TAAC	*Tamarix canariensis*
ERIBO	*Conyza bonariensis*	VITVI	*Vitis vinifera*
ERISU	*Conyza sumatrensis*		

**Table 3:** Field trials characteristics.

Field Trial	Vine	Vineyard Establishment	Coordinates ETRS89		Spacing (m)		Soil Texture (%)			pH	(%)
	Variety		Latitude	Longitude	Between	Within	Sand	Silt	Clay		*O.M.
Trial 1	Cabernet Sauvignon	2009	41°39’28.1”N	0°31’11.3”E	3.0	1.5	28.4	47.7	24.2	8.40	1.61
Trial 2			41°39’30.7”N	0°31’13.8”E							
Trial 3	Pinot Noir	2010	41°40’28.8”N	0°28’00.0”E	2.8	1.5	27.9	38.9	33.2	8.18	2.32

*O.M.: Organic matter.

## Supplementary Materials

The following are available online at https://www.mdpi.com/article/10.3390/plants11202785/s1,Table S1: Signif-icance of the one-way ANOVA test for Trial 1. F and *p* values of each sampling date, Table S2: Significance of the one-way ANOVA test for Trial 2. F and *p* values of each sampling date.

## References

[1] MAPA (2021). Encuesta Sobre Superficies Y Rendimientos de Cultivos. Resultados Nacionales Y Autonómicos. Subsecretaría De Agricultura, Pesca Y Alimentación. Subdirección General de Análisis, Coordinación y Estadística. https://www.mapa.gob.es/es/estadistica/temas/estadisticas-agrarias/comentariosespana2021_tcm30-584074.pdf.

[2] Oerke E.C. (2006). Crop losses to pests. J. Agric. Sci..

[3] Byrne M.E., Howell G.S. (1978). Initial response of Baco Noir grapevines to pruning severity, sucker removal, and weed control Amer. J. Enol. Viticult..

[4] Hembree K.J., Lanini W.T. (2006). Weeds. UC IPM Pest Management Guidelines: Grape.

[5] MAPA (2019). Encuesta Sobre Superficies y Rendimientos De Cultivos. Análisis De Las Plantaciones De Viñedo En España Subsecretaría De Agricultura, Pesca Y Alimentación. Subdirección General de Análisis, Coordinación y Estadística. https://www.mapa.gob.es/es/estadistica/temas/estadisticas-agrarias/vinedo2019_tcm30-562250.pdf.

[6] Guerra B., Steenwerth K. (2011). Influence of Floor Management Technique on Grapevine Growth, Disease Pressure, and Juice and Wine Composition: A Review. Am. J. Enol. Vitic..

[7] Narayan S., Liew Z., Bronstein J.M., Ritz B. (2017). Occupational pesticide use and Parkinson’s disease in the Parkinson Environment Gene (PEG) study. Environ. Int..

[8] Tourte L., Smith R., Bettiga L., Bensen T., Smith J., Salm D. (2008). Post-emergence herbicides are cost effective for vineyard floor management on the Central Coast. Calif. Agric..

[9] Haring S.C., Ou J., Al-Khatib K., Hanson B.D. (2022). Grapevine Injury and Fruit Yield Response to Simulated Auxin Herbicide Drift. HortScience.

[10] Zaller J.G., Cantelmo C., Dos Santos G., Muther S., Gruber E., Pallua P., Mandi K., Friedrich B., Hofstetter I., Schmuckenschlager B. (2018). Herbicides in vineyards reduce grapevine root mycorrhization and alter soil microorganisms and the nutrient composition in grapevine roots, leaves, xylem sap and grape juice. Environ. Sci. Pollut. Res. Int..

[11] Bajwa A.A., Sadia S., Ali H.H., Jabran K., Peerzada A.M., Chauhan B.S. (2016). Biology and management of two important *Conyza* weeds: A global review. Environ. Sci. Pollut. Res..

[12] Urbano J.M., Borrego A., Torres V., León J., Jimenez C., Dinelli G., Barnes J. (2007). Glyphosate-resistant hairy fleabane (*Conyza bonariensis*) in Spain. Weed Technol..

[13] Alcorta M., Fidelibus M.W., Steenwerth K.L., Shrestha A. (2011). Competitive Effects of Glyphosate-Resistant and Glyphosate-Susceptible Horseweed (*Conyza canadensis*) on Young Grapevines (*Vitis vinifera*). Weed Sci..

[14] Heap I., Duke S.O. (2018). Overview of glyphosate-resistant weeds worldwide. Pest Manag. Sci..

[15] Harvey M., Pilgrim S. (2011). The new competition for land: Food, energy, and climate change. Food Policy..

[16] MAPA (2021). Producción ecológica. Estadísticas 2020. Subdirección General de la Calidad Alimentaria y de Laboratorios Agroalimentarios. https://www.mapa.gob.es/es/alimentacion/temas/produccion-eco/estadisticas_pe_2020_tcm30-564465.pdf.

[17] Kloen H., Daniels L. (2000). Onderzoeksagenda Biologische Landbouw & Voeding 2000-2004. Biologica/Wageningen UR.

[18] Cerdan O., Govers G., Le Bissonnais Y., Van Oost K., Poesen J., Saby N., Gobin A., Vacca A., Quinton J., Auerswald K. (2010). Rates and spatial variations of soil erosion in Europe: A study based on erosion plot data. Geomorphology..

[19] Prosdocimi M., Cerdà A., Tarolli P. (2016). Soil water erosion on Mediterranean vineyards: A review. Catena..

[20] Lanini W.T., McGourty G.T., Thrupp L.A., McGourty G. (2011). Weed management for organic vineyards. Organic Winegrowing Manual.

[21] Smart D.R., Schwass E., Lakso A., Morano L. (2006). Grapevine rooting patterns: A comprehensive analysis and a review. Am. J. Enol. Viticult..

[22] Abad J., Hermoso De Mendoza I., Marín D., Orcaray L., Santesteban L.G. (2021). Cover crops in viticulture. A systematic review (1): Implications on soil characteristics and biodiversity in vineyard. OenoOne.

[23] Glover J.D., Reginald J.P., Andrews R.K. (2000). Systematic method for rating soil quality of conventional, organic, and integrated apple orchards in Washington state. Agric. Ecosyst. Environ..

[24] Smith R., Bettiga L., Cahn M., Baumgartner K., Jackson L.E., Bensen T. (2008). Vineyard floor management affects soil, plant nutrition, and grape yield and quality. Calif. Agric..

[25] Virto I., Imaz M.J., Fernández-Ugalde O., Urrutia I., Enrique A., Bescansa P. (2012). Soil quality evaluation following the implementation of permanent cover crops in semi-arid vineyards. Organic matter, physical and biological soil properties. Span. J. Agric. Res..

[26] Jradi S., Bouzdine T., Bernard Delhomme C., Jaegler A. (2018). Tracking carbon footprint in French vineyards: A DEA performance assessment. J. Cleaner Product..

[27] Bàrberi P. (2002). Weed management in organic agriculture: Are we addressing the right issues?. Weed Res..

[28] MacLaren C., Storkey J., Menegat A., Metcalfe H., Dehnen-Schmutz K. (2020). An ecological future for weed science to sustain crop production and the environment. A review. Agron. Sustain. Dev..

[29] Bond W., Grundy A.C. (1995). Non-chemical weed management in organic farming systems. Weed Res..

[30] Steinmaus S., Elmore C.L., Smith R.J., Donaldson D., Webber A., Roncoroni J., Miller P.R.M. (2008). Cover crops as an alternative to conventional weed management systems in vineyards. Weed Res..

[31] Elmore C.L., Donaldson D.R., Smith R.J., Ingels C.A. (1998). Weed management Cover Cropping in Vineyards.

[32] Dhima K.V., Vasilakoglou I.B., Eleftherohorinos I.G., Lithourgidis A.S. (2006). Allelopathic Potential of Winter Cereals and Their Cover Crop Mulch Effect on Grass Weed Suppression and Corn Development. Crop Sci..

[33] Davies W.J., Zhang J., Yang J., Dodd I.C. (2011). Novel crop science to improve yield and resource use efficiency in water-limited agriculture. J. Agric. Sci..

[34] Myburgh P.A. (2013). Effect of shallow tillage and straw mulching on soil water conservation and grapevine response. S. Afr. J. Plant Soil..

[35] Buesa I., Mirás-Avalos J.M., De Paz J.M., Visconti F., Sanz F., Yeves A., Guerra D., Intrigliolo D.S. (2021). Soil management in semi-arid vineyards: Combined effects of organic mulching and no-tillage under different water regimes. Eur. J. Agron..

[36] Linares Torres R., De La Fuente Lloreda M., Junquera Gonzalez P., Lissarrague García-Gutierrez J., Baeza Trujillo P. (2018). Effect of soil management strategies on the characteristics of the grapevine root system in irrigated vineyards under semi-arid conditions. Aus. J. Grape Wine Res..

[37] López-Urrea R., Sánchez J.M., Montoro A., Mañas F., Intrigliolo D.S. (2020). Effect of using pruning waste as an organic mulching on a drip-irrigated vineyard evapotranspiration under a semi-arid climate. Agric. For. Meteorol..

[38] Varga P., Májer J. (2004). The use of organic wastes for soil-covering of vineyards. Acta Hortic..

[39] Thomson L.J., Hoffmann A.A. (2007). Effects of ground cover (straw and compost) on the abundance of natural enemies and soil macro invertebrates in vineyards. Agric. Forest Entomol..

[40] Mundy D.C., Agnew R.H. (2002). Effects of mulching with vineyard and winery waste on soil fungi and Botrytis bunch rot in Marlborough vineyards. N.Z. Plant Protec..

[41] EPPO (2022). European and Mediterranean Plant Protection Organization Global Database. https://gd.eppo.int.

[42] Teasdale J.R., Mohler C.L. (1993). Light transmittance, soil temperature and soil moisture under residue of hairy vetch and rye. Agron. J..

[43] White R.H., Worsham A.D., Blum U. (1989). Allelopathic potential of legume debris and aqueous extracts. Weed Sci..

[44] Moonen A.C., Barberi P. (2006). An ecological approach to study the physical and chemical effects of rye cover crop residues on Amaranthus retroflexus, Echinochloa crus-galli and maize. Ann. Appl. Biol..

[45] Brown S.M., Whitwell T. (1988). Influence of tillage on horseweed (Conyza canadensis). Weed Tech..

[46] Wicks G.A., Felton W.L., Murison R.D., Martin R.J. (2020). Changes in fallow weed species in continuous wheat in northern New SouthWales, 1981–1990. Animal Prod. Sci..

[47] Heap I. (2008). The International Herbicide-Resistant Weed Database. http://weedscience.org/Home.aspx.

[48] Loura D., Sahil Florentine S., Chauhan B.S. (2020). Germination ecology of hairy fleabane (*Conyza bonariensis*) and its implications for weed management. Weed Sci..

[49] Wu H., Walker S., Rollin M.J., Tan D.K.Y., Robinson G., Werth J. (2007). Germination, persistence, and emergence of flaxleaf fleabane (*Conyza bonariensis* [L.] Cronquist). Weed Biol Manage..

[50] Benvenuti S., Macchia M. (1995). Hypoxia effect on buried weed seed germination. Weed. Res..

[51] Tebeau A.S., Alston D.G., Ransom C.V., Black B.L., Reeve J.R., Culumber C.M. (2017). Effects of floor vegetation and fertility management on weed biomass and diversity in organic peach orchards. Weed Technol..

[52] Abad J., Marín D., Santesteban L.G., Cibriain J.F., Sagüés A. (2020). Under-vine cover crops: Impact on weed development, yield and grape composition. Oeno One.

[53] Ormeño-Núñez J., Pino-Rojas G., Garfe-Vergara F. (2008). Inhibition of yellow nutsedge (Cyperus esculentus L.) and bermudagrass (Cynodon dactylon (L.) Pers.) by a mulch derived from rye (Secale cereale L.) in grapevines. Chil. J. Agric. Res..

[54] Valencia-Gredilla F., Royo-Esnal A., Juárez-Escario A., Recasens J. (2020). Different Ground Vegetation Cover Management Systems to Manage Cynodon dactylon in an Irrigated Vineyard. Agronomy..

[55] Teasdale J.R., Mohler C.L. (2000). The quantitative relationship between weed emergence and the physical properties of mulches. Weed Sci..

[56] Gallagher R.S., Cardina J. (1998). Phytochrome-mediated Amaranthus germination II: Development of very low fluence sensitivity. Weed Sci..

[57] Bartley P.C., Wehtje G.R., Murphy A.M., Foshee W.G., Gilliam C.H. (2017). Mulch type and depth influences control of three major weed species in nursery container production. HortTechnology.

[58] Teasdale J.R., Beste C.E., Potts W.E. (1991). Response of weeds to tillage and cover crop residue. Weed Sci..

[59] Bremer E., van Houtum W., van Kessel C. (1991). Carbon dioxide evolution from wheat and lentil residues as affected by grinding, added nitrogen, and the absence of soil. Biol. Fertil. Soils..

[60] Sims J.L., Frederick L.R. (1970). Nitrogen immobilization and decomposition of corn residue in soil and sand as affected by residue particle size. Soil Sci..

[61] Goh K.M., Tutua S.S. (2004). Effects of Organic and Plant Residue Quality and Orchard Management Practices on Decomposition Rates of Residues. Commun. Soil Sci. Plant Analysis..

[62] Fredrikson L., Skinkis P.A., Peachey E. (2011). Cover Crop and Floor Management Affect Weed Coverage and Density in an Establishing Oregon Vineyard. HortTechnology.

[63] DeVetter L.W., Dilley C.A., Nonnecke G.R. (2015). Mulches reduce weeds, maintain yield, and promote soil quality in a continental-climate vineyard. Am. J. Enol. Viticult..

[64] Fraga H., Santos J.A. (2018). Vineyard mulching as a climate change adaptation measure: Future simulations for Alentejo, Portugal. Agric. Syst..

[65] Morlat R., Chaussod R. (2008). Long-term additions of organic amendments in a Loire Valley vineyard. I. Effects on properties of a calcareous sandy soil. Am. J. Enol. Vitic..

[66] García-Díaz A., Marqués M.J., Sastre B., Bienes R. (2018). Labile and stable soil organic carbon and physical improvements using groundcovers in vineyards from central Spain. Sci. Total Environ..

[67] Colugnati G., Cattarossi G., Crespan G. (2004). Gestione del terreno in viticoltura. Vigne Vini..

[68] Fourie J.C. (2011). Soil managemetn in the Breede River Valley wine grape region, South Africa. 3. Grapevine performance. S. Afr. J. Enol. Vitic..

[69] Manzone M., Demeneghi M., Marucco P., Grella M., Balsari P. (2020). Technical solutions for under-row weed control in vineyards: Efficacy, costs and environmental aspects analysis. J. Agric. Eng..

[70] Abad J., Hermoso De Mendoza I., Marín D., Orcaray L., Santesteban L.G. (2021). Cover crops in viticulture. A systematic review (2): Implications on vineyard agronomic performance. OenoOne.

[71] Ross O.C. (2010). Reflective Mulch Effects on the Grapevine Environment, Pinot Noir Vine Performance and Juice and Wine Characteristics. Master’s Thesis.

[72] Kottek M., Grieser J., Beck C., Rudolf B., Rubel F. (2006). World Map of the Köppen-Geiger climate classification updated. Meteorol..

