# Supplementary material for: Organic Mulches as an Alternative to Conventional Under-Vine Weed Management in Mediterranean Irrigated Vineyards
Source: Plants (Basel). 2022 Oct 20;11(20):2785. doi: 10.3390/plants11202785 (PMC9608967; doi:10.3390/plants11202785)
Supplement: Supplementary file 1 [file plants-11-02785-s001.zip › plants-1876447-supplementary.pdf]

Table S1. Significance of the one-way ANOVA test for Trial 1. F and *p* values of each sampling date.

Trial 1			
Date		F	*p*
2017	March	4,728	0,0211
	May	3,208	0,0587
	July	2,714	0,0913
	September	3,0892	0,0684
2018	March	4,4285	0,0257
	May	2,9286	0,0766
	July	3,4361	0,0516
	September	8,1981	0,0034
2019	April	18,5049	0,0001

Table S2. Significance of the one-way ANOVA test for Trial 2. F and p values of each sampling date.

Trial 2			
Date		F	*p*
2017	May	4,1589	0,0057
	July	12,5561	<0,0001
2018	May	85,6948	<0,0001
	July	16,2971	<0,0001
	August	10,4635	<0,0001
2019	April	23,2979	<0,0001
